# Infant Pneumonitis Due to a Tracheoesophageal Fistula: A Presentation of Two Autopsy Cases

**DOI:** 10.7759/cureus.49959

**Published:** 2023-12-05

**Authors:** Athina Tousia, Ioannis Platzas, Nikolaos Goutas, Dimitrios Vlachodimitropoulos, Konstantinos Katsos, Christoforos Kolentinis, Maria Piagkou, Emmanouil I Sakelliadis

**Affiliations:** 1 Forensic Medicine and Toxicology, National and Kapodistrian University of Athens School of Medicine, Athens, GRC; 2 Anatomy and Surgical Anatomy, National and Kapodistrian University of Athens School of Medicine, Athens, GRC

**Keywords:** infants, esophageal atresia, tracheoesophageal fistula, aspiration pneumonia, aspiration pneumonitis, death, autopsy

## Abstract

Both esophageal atresia (EA) and tracheoesophageal fistula (TEF) represent a rather uncommon congenital abnormality that is the result of abnormal tracheoesophageal organogenesis. Although EA, with or without TEF, is relatively uncommon, it represents the most common upper gastrointestinal birth defect. Esophageal atresia and tracheoesophageal fistula are anatomically classified into five types according to the Gross classification (types A, B, C, D, E/H). As in type E/H, the continuity of the esophagus is not interrupted, the symptom onset is consequently delayed, and therefore diagnosis is difficult.

Aspiration pneumonitis is a chemical injury caused by inhaled sterile gastric contents, while aspiration pneumonia is, in part, an infectious process because the inhaled oropharyngeal secretions are rich in bacteria. This paper aims to report two infant autopsy cases of aspiration pneumonitis with TEF involvement. The main histopathological finding was interstitial pneumonitis. Upon histopathological examination, lymphocytes, plasma cells, and macrophages were discovered on the alveolar walls, which were compatible with the chemical origin of interstitial pneumonitis. No eosinophils were detected; therefore, hypersensitivity-originating interstitial pneumonitis was ruled out. The cause of death in both cases was interstitial pneumonitis.

## Introduction

Cases in which esophageal atresia (EA) and/or tracheoesophageal fistula (TEF) are diagnosed during post-mortem examination are rare. As such, it is important for forensic pathologists to properly describe their findings and publish relevant reports to raise awareness for this rare pathology that may lead to infant death.

The inhalation of either oral or gastric contents in the lower respiratory tract is called aspiration [1]. Its effect varies according to the origin of the material aspirated (pharynx or stomach), its fluidity and pH, the possible presence of bacteria, and, essentially, the aspiration’s volume and chronicity. Aspiration pneumonitis (Mendelson’s syndrome) is a chemical injury caused by inhaled sterile gastric contents, while aspiration pneumonia is, in part, an infectious process because the inhaled oropharyngeal secretions are rich in bacteria. [2]. Gastric content-related-aspiration pneumonia is present in more than one-third of lung pathology findings of infants whose deaths are attributed to sudden infant death syndrome (SIDS) [3]. Relevant symptom severity varies depending mainly on the aspirated material (nature and quantity). The most commonly described symptoms may include tachypnoea, increased heart rate, cough, increased body temperature, fatigue, muscle pain, loss of weight, malaise, end-expiratory wheezing, decreased oxygen in the systemic circulation, chest pain (pleuritic), bronchoconstriction, and pulmonary edema [3].

Both EA and/or TEF represent a rather uncommon congenital abnormality that is the result of abnormal tracheoesophageal organogenesis [4]. Both the trachea and esophagus develop from a common primitive foregut. At about the fourth gestational week, both the respiratory and gastrointestinal tracts are divided by epithelial ridges. Consequently, a ventral respiratory tract and a dorsal esophageal tract are formed. The fistula formation is believed to arise from an embryonic lung bud that did not undergo branching [5, 6]. Esophageal atresia, with or without TEF, is uncommon [7]. Nevertheless, it is the most common upper gastrointestinal birth defect. Most cases of EA/TEF (~50-70) are associated with other major birth defects. The mechanism behind non-syndromic EA/TEF remains unclear, although a wide range of environmental and genetic factors have been proposed in the literature [6]. The incidence of TEF is reported to be one in 3,500 births [6, 8]. The mortality rates of EA/TEF were superior in infants with associated cardiac conditions (42% versus 12% without) [6]. Gastrointestinal conditions, including gastroesophageal reflux disease (GERD), esophageal dysmotility, strictures, eosinophilic esophagitis (EoE), and tracheomalacia [9], are often etiologically involved. Esophageal atresia/tracheoesophageal fistulas are anatomically classified into five types according to the Gross classification (types A, B, C, D, E/H) [10, 11]. In type A (~7%), only EA exists; no fistula is present. In type B, an EA with proximal TEF exists. In type C (most frequent, ~70%), an EA with a distal TEF is present. In type D, an EA with both proximal and distal fistulas exists. Finally, in type E, also known as "type H,” only a TEF is noted, without the presence of EA. Types B, D, and E/H are rare [7,11]. The H-type fistula occurs in only 4% of cases [12]. The differential diagnosis of TEF/EA may include, among others, severe GERD, either esophageal stricture or esophageal diverticulum, webs, esophageal duplication, esophageal perforation (iatrogenic), congenital short esophagus, cleft, and agenesis or atresia of the trachea [6,12]. The clinical diagnosis of EA consists of the insertion of an orogastric or nasogastric catheter, a CT scan, water-soluble contrast instillation under fluoroscopic guidance, esophageal endoscopy/bronchoscopy and the injection of methylene blue into the trachea [13, 14]. Frequently, E/H-type TEF can be difficult to diagnose since the continuity of the esophagus is not interrupted; therefore, the appearance of symptoms is delayed and the diagnosis is often missed [6]. Aspiration is confirmed by histopathological examination of the lung demonstrating either evidence of aspirated material or foreign body granulomas [1]. Bronchoalveolar lavage (BAL) may also be used to find the presence of lipid-laden macrophages [1]. The initial treatment of choice to prevent aspiration syndromes is conservative therapy [15]. After conservative therapy, the treatment of choice is surgical intervention [16].

## Case presentation

Case one

An approximately 50-day-old male infant was found pulseless by his mother with vomit and blood on his cheek, during the early morning hours. His last meal was four hours before. No feeding history problems were reported. The infant was brought to the emergency department (ED). At the time of the clinical examination, the infant was pulseless, apneic, and acrocyanotic, with torso pallor, mydriasis, and blood effusing from the nasal cavity. After an initial assessment and first aid treatment at the ED, the infant was admitted to the ICU. During the first day of hospitalization, the infant presented with cardiopulmonary arrest, and both cardiopulmonary resuscitation (CPR) and intubation were performed without any result. The infant was a firstborn conceived by in-vitro fertilization and was delivered via cesarean delivery at the 35th week of gestation due to polyhydramnios and non-stress test alterations suggesting fetal distress. After birth (birth weight: 2.5 kg), the newborn remained in a neonatal ICU due to prematurity for four days. Thereafter, no problem was reported.

**Figure 1:**
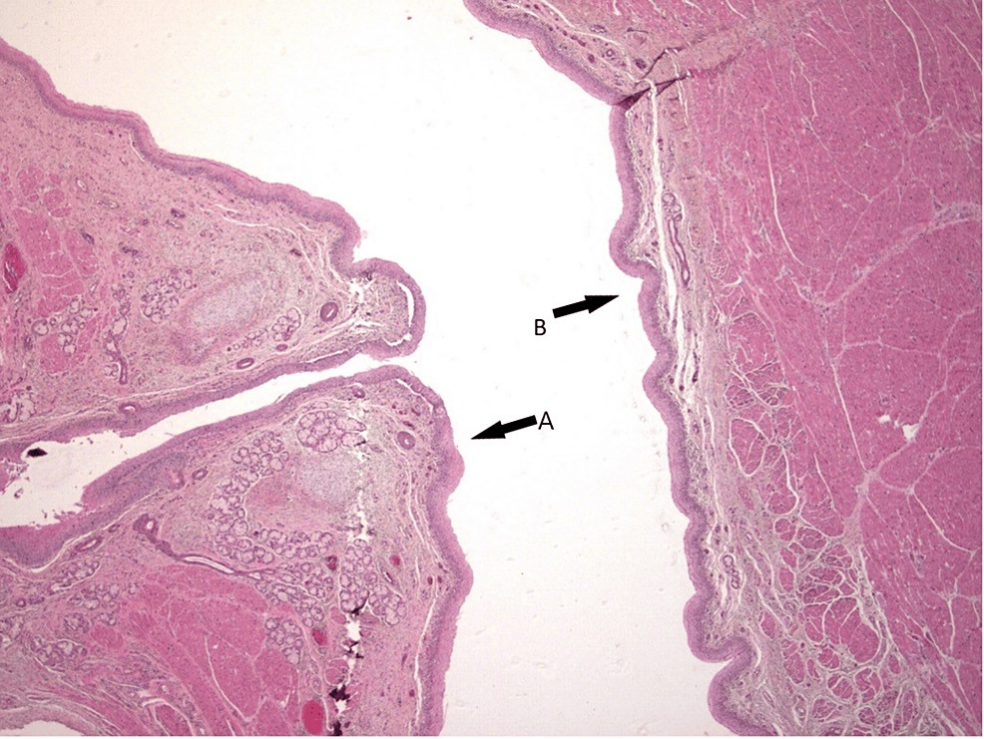
Case one: the H&E staining reveals a tracheoesophageal fistula (x25). (A) The respiratory epithelium (trachea) is observed on the left. (B) The squamous epithelium of the esophagus is observed in the center.

The deceased infant was submitted to a post-mortem examination. During the autopsy, no evident macroscopical finding was discovered. No external birth defects were noted. Histopathological examination of the brain was free of any findings. Thymus histopathological findings were compatible with hypoxia. A type H TEF was noted upon esophageal examination (Figure 1).

Both lungs presented severe congestion, edema, extensive pulmonary hemorrhage, and severe interstitial pneumonitis (dense lymphocytic, plasmatocytic, and macrophage infiltration were observed). The heart weight was measured to be 25 grams. The heart examination did not reveal any pathological findings, other than interstitial edema. Based on the histopathologic evaluation of tissue samples obtained, death was attributed to interstitial pneumonitis.

Case two

A female infant about two months old was brought to the ED during the early afternoon hours by her parents and was directly admitted to the ICU. No other information about the medical history of the infant was available. Both CPR and intubation were performed without any results. Laboratory examinations revealed proBNP at 30,300 pg/mL, troponine at 54 ng/L, mild transaminase elevation, and absence of infection (viral screening and C-reactive protein (CRP) testing were negative). Information provided by the parents suggested a recent change in the milk given to the infant, which resulted in significant gas production thereafter. According to the treating physicians, death was attributed possibly to supraventricular tachycardia, cardiogenic shock, bradycardia, and sudden onset pulmonary edema.

**Figure 2:**
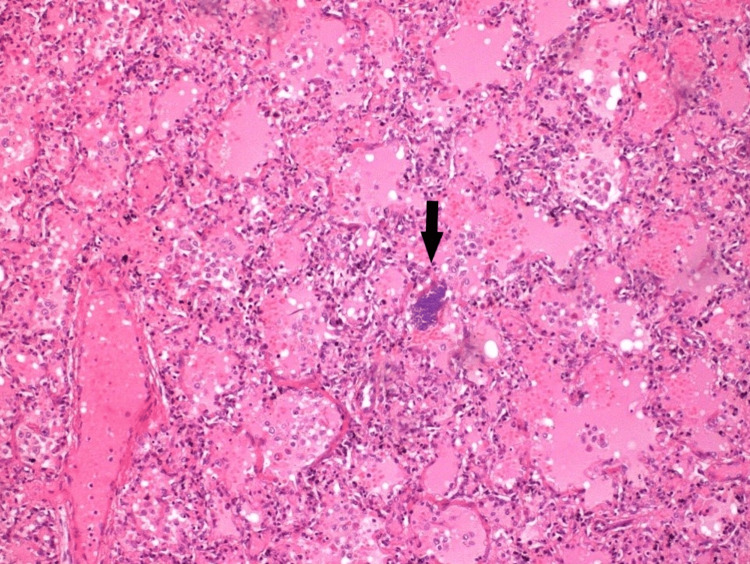
Case two: the H&E staining revealed aspiration pneumonitis (x100). Congestive pulmonary tissue with medium to severe inflammation was seen. On the center (indicated by the arrow), basophilic material (foreign body or food particle) is observed.

The deceased infant was submitted for a post-mortem examination. During the autopsy, no evident macroscopical finding was discovered. No external birth defects were noted. Histopathological examination of the brain revealed only hypoxia-compatible findings (red neurons). The thymus presented hypoxic changes. A type H tracheoesophageal fistula was ascertained during the esophageal examination. Both lungs presented severe interstitial pneumonitis (Figure 2).

Thickening of the alveolar walls, increased infiltration by macrophages and lymphocytes, and thickening of the walls of small intraparenchymal vessels (due to pulmonary hypertension) were also observed. The heart weight was measured to be 25 grams, without any defects. The heart examination did not reveal any pathological findings other than interstitial edema. Again, based on the histopathologic evaluation of the tissue samples obtained, death was attributed to interstitial pneumonitis.

Toxicological analysis revealed midazolam and lidocaine, both in therapeutic concentrations because of the in-hospital administration.

## Discussion

Both infants’ pulmonary histopathological findings suggested interstitial pneumonitis as well as an H-type TEF. According to the literature, aspiration is confirmed by a histopathological examination of the lung demonstrating either evidence of aspirated material or foreign body granulomas [1]. However, no gold standard to differentiate between aspiration pneumonitis and pneumonia exists [17]. Nevertheless, no infection-compatible findings were observed in both histopathological examinations, thus indirectly suggesting the development of pneumonitis was caused by aspiration. 

Most cases of EA/TEF (~50-70) are associated with other major birth defects. In both cases examined, no external birth defects were noted. Multiple chromosomal or single-gene syndromes are linked to EA/TEF. Such abnormalities may include trisomies (21 and 18), the CHARGE syndrome, and VACTERL or VATER association [6, 11]. The CHARGE syndrome is characterized by eye coloboma, heart defects, choanal atresia, developmental retardation, hypoplasia of the genitals, and finally, ear defects [6,11]. The VACTERL, or VATER association, refers to defects of the vertebrae, atresia of the anus, cardiac defects, TEF, and renal and limb abnormalities [6,11]. The non-syndromic EA/TEF mechanism remains yet to be clarified, although a wide range of environmental and genetic factors have been proposed throughout the literature [6].

The inability to feed orally within the first month of life, combined with an increased incidence of gastrointestinal and respiratory problems, is a predictor of poor outcomes [7,18]. In the cases discussed, no feeding issues were reported by the parents. 

Patients who present with EA/TEF tend to have considerable growth deficiencies [6]. After adjustment, every 100-gram increase in body weight decreased mortality risk by approximately 11% [19]. Both infants’ heart weight was normal and compatible with their weight centiles [20].

As stated above, H-type TEF can be difficult to diagnose since it can be asymptomatic at first; therefore, the diagnosis may often be delayed [6]. The clinical diagnosis of EA consists of the insertion of an orogastric or nasogastric catheter (inability to pass after 10-15 cm), a CT scan, water-soluble contrast instillation into the esophageal pouch under fluoroscopic guidance, esophageal endoscopy/bronchoscopy and the injection of methylene blue into the trachea. It is important to know that barium contrast should be avoided, as in cases of aspiration, it is associated with pneumonitis [13,14]. Both infants presented symptoms of acute respiratory distress. The management of an acute aspiration event consists of symptomatic and supportive management, which includes oxygenation, ventilation, improvement in persistent pulmonary hypertension, and maintenance of systemic circulation [15]. Surgical intervention is mandatory. Standard surgical preparation for such cases, according to the literature, requires the patient to be weaned off mechanical ventilation and to correct any other respiratory pathology (e.g., to treat any co-existing respiratory infection). Physiotherapy is often required, while malnutrition should be corrected through enteral feeding. As the surgical procedure is challenging, increased morbidity and mortality are expected. However, since it may lead to outstanding long-term results, it is considered to be the first line of treatment for TEF [16].

A macroscopical diagnosis of a TEF is quite difficult during a post-mortem examination. As such, it poses a challenge that may be overcome with the help of a histopathological examination that can identify even the smallest lesions. Extensive tissue sampling allows the discovery of a wide range of pathologies in all organs and is almost always required. If the patient's history includes feeding disorders, a forensic pathologist should be alert to identify the fistula macroscopically, if possible, or to proceed with adequate tissue sampling. A referral to a histopathologist specializing in and experienced in neonatal pathology is probably the best approach to guarantee optimal handling and reliable results.

## Conclusions

Aspiration pneumonitis is a rare clinical condition but very common among infants with TEF. Early endoscopic and radiographic diagnosis, along with surgical intervention and comorbidity management, may improve the outcome by reducing the possibility of complications such as aspiration pneumonitis. Forensic pathologists must identify and report these kinds of cases in order to raise awareness of the condition, its findings, and its resolution.
